# Evaluation of the Diagnostic Performance of Recombinant Antigen B1 for Detection of Cystic Echinococcosis Using Lateral Flow Dipstick Test

**DOI:** 10.18502/ijpa.v15i3.4191

**Published:** 2020

**Authors:** Rahmah NOORDIN, Sam KHANBABAIE, Muhammad HAFIZNUR YUNUS, Hanspeter MARTI, Beatrice NICKEL, Majid FASIHI HARANDI, Saeid NASIBI

**Affiliations:** 1. Institute for Research in Molecular Medicine (INFORMM), Universiti Sains Malaysia, 11800 USM, Pulau Pinang, Malaysia; 2. Swiss Tropical and Public Health Institute, Basel, Switzerland; 3. University of Basel, Basel, Switzerland; 4. Research Center for Hydatid Disease in Iran, School of Medicine, Kerman University of Medical Sciences, Kerman, Iran

**Keywords:** Rapid test, Lateral flow, Cystic and alveolar echinococcosis, Evaluation, Immunoglobulin G

## Abstract

**Background:**

Human echinococcosis is a neglected zoonotic disease distributed worldwide. It comprises cystic and alveolar forms, the former being the more prevalent disease. Imaging techniques are the first choice for diagnosis of cystic echinococcosis and serology is used as an additional diagnostic technique in doubtful cases or as the sole test in low-resource settings. Rapid diagnostic tests are useful and convenient for immunodiagnosis of cystic echinococcosis in endemic areas, where medical facilities often struggle with limited resources.

**Methods:**

Recently, we have developed Hyd Rapid™, an IgG4 lateral flow dipstick test using recombinant antigen B1 for detection of cystic echinococcosis. This study was performed between 2016 until 2018 at the Institute for Research in Molecular Medicine, Universiti Sains Malaysia. The diagnostic performance of Hyd Rapid™ was tested in-house and at two international laboratories in Switzerland and Iran.

**Results:**

The overall diagnostic sensitivity for detection of cystic and alveolar echinococcosis was 95% (56/59). Meanwhile, the diagnostic specificity, with and without exclusion of cysticercosis and fascioliasis, was 100% (n=48) and 88% (63/72), respectively.

**Conclusion:**

Hyd Rapid™ detected cystic echinococcosis as well as probable cases of alveolar echinococcosis. Therefore, Hyd Rapid™ showed good potential as a serological tool for echinococcosis, and merits further evaluation.

## Introduction

Human cystic echinococcosis (CE) or hydatid cyst disease is a zoonosis caused by infection with the larval stage of the dog tapeworm *Echinococcus granulosus sensu lato,* which mainly occurs in pastoral areas (1,2). CE is distributed worldwide and causes economic loss (3–5). The infection is asymptomatic even up to several years after infection, so early diagnosis, especially during the asymptomatic period, is important for the appropriate management and early treatment of the disease to reduce morbidity and mortality (6). Imaging techniques are preferred as the primary tool for the diagnosis of CE. However, the small size and location of some cysts may pose a diagnostic challenge if a physician relies only on imaging (7,8).

Serology is used as a confirmatory test and may be useful for CE diagnosis in low-resource and remote areas since it is easier and cheaper than imaging methods (9). There are limitations of the available serological assays including cross-reactivity with other parasitic infections, and variable diagnostic sensitivity due to cyst characteristics (location, stage, size, and the number of cysts) and antigen type (10).

There are several reports on the development of CE diagnostic assays using various protein antigens and different techniques (11–13). The most common antigen source for CE immunodiagnosis is hydatid cyst fluid (HCF), with antigen B and 5 being the main antigenic components in the assays (11,14). Some studies recorded good performance in detecting CE using the abovementioned native antigens in various formats (15–19). However, using native antigens for the manufacturing of these tests would be a challenge due to the issues of upscaling, standardization, and batch-to-batch variation. So, using recombinant antigens may be a solution; furthermore, recombinant antigens may increase the performance of the test, especially in reducing non-specific reactions (11,14). In particular, recombinant AgB showed good diagnostic sensitivity and specificity, thus a promising candidate for the development of antibody detection assays, including rapid diagnostic tests (RDT), for CE (11,20,21).

Previously, an IgG4 lateral flow dipstick test was developed using native AgB to detect specific human antibodies against CE (22). It showed 95% diagnostic sensitivity and 100% specificity with the serum panel used in the study. Our laboratory also introduced an antigen detection test in the format of lateral flow dipstick for the diagnosis of echinococcosis (23). As a follow-up, the present study describes the development of a lateral flow IgG4 dipstick test using recombinant antigen B1 (rAgB) to detect CE called Hyd Rapid™.

This study presents results of an evaluation of the diagnostic performance of Hyd Rapid™ i.e., in-house at Universiti Sains Malaysia (USM) and two international diagnostic centers, namely Swiss Tropical and Public Health Institute (Swiss TPH) and Kerman University of Medical Sciences, Iran (KMU).

## Materials and Methods

### Samples

The diagnostic performance of Hyd Rapid™ was first evaluated in-house at USM using serum samples from Iranian patients with surgically-confirmed CE collected from different hospitals in Tehran, Iran in year 2014 and brought to USM (n = 21), healthy individuals from Malaysia (non-endemic for echinococcosis) (n = 11), and samples from patients in Malaysia with other helminth infections (n = 32) i.e. fascioliasis (n = 3), schistosomiasis (n = 7), cysticercosis (n = 1), taeniasis (n = 1), ascariasis (n=7), trichuriasis (n=8), and hookworm (n=5). Except for cysticercosis, in which the laboratory diagnosis was performed by serology, all other infections were diagnosed parasitologically. Hyd Rapid™ was then independently tested for its diagnostic sensitivity and specificity at two international diagnostic centers i.e., Swiss TPH, Basel, Switzerland, and KMU, Kerman, Iran.

A total of 50 serum samples sent to Swiss TPH from various local hospitals in Basel, were tested at the institute. For sensitivity testing, sera of patients infected with *E. granulosus* (n=10) and probable *E. multilocularis* (n=10) were used. *E. granulosus* sera were classified by a positive *E. granulosus*-in house ELISA (using hydatid fluid antigen), a positive *E. granulosus* in-house indirect hemagglutination assay (IHA), a positive EITB for *E. granulosus* (LDBio Diagnostics, France), a negative Em2-Em18-ELISA (Bordier affinity products SA, Switzerland), and presence of liver cysts. Probable cases of *E. multilocularis* sera were classified by a positive Em2-Em18-ELISA, a positive EMG11/Em18 ELISA, a positive EITB for *E. multilocularis* (LDBio Diagnostics), and presence of liver lesions. For determination of specificity, sera of healthy blood donors (n=5) and sera of the following infections were tested: *Taenia solium* cysticercosis (n=10), *Fasciola hepatica* (n=11), *Schistosoma mansoni* (n=2) and *Strongyloides stercoralis* (n=2). Sera of these infections were classified by parasitological confirmation and/or positive in-house serology tests at the Diagnostic Centre of the institute.

At KMU, the diagnostic sensitivity was assessed using serum samples from surgically confirmed CE patients with active hydatid cyst lesions, including nine samples from patients with lung CE and ten samples from patients with liver CE. These samples belonged to local patients and were collected from various hospitals in Kerman, Iran. Before the surgery, the patients underwent CT and ultrasound scans. Moreover, 40 serum samples from apparently healthy local blood donors were tested.

The evaluations were performed by the respective laboratory personnel and their respective research Ethics Committees approved the use of banked/stored anonymized serum samples.

### Hyd Rapid™

**Fig. 1:**
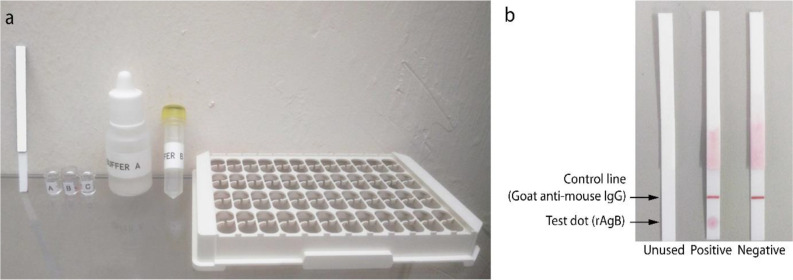
Prototype Hyd Rapid^TM^ test. a) Components of Hyd Rapid^TM^ test; from left: a dipstick strip, a triplet well unit, Chase Buffer (buffer A), PBS buffer (buffer B) and well holder. b) Test strips of Hyd Rapid^TM^ showing an unused strip, a positive and negative result

The prototype Hyd Rapid™ contains the following components (Fig. 1a): a dipstick strip, a detachable triplet well unit that is inserted in a flat-bottomed microplate (function as a holder for the triplet well unit), Buffer A (phosphate-buffered saline, pH 7.2) and Buffer B (commercially available as ‘Chase Buffer,’ Reszon Diagnostics International, Selangor, Malaysia).

The dipstick strip comprised rAgB in the test dot position and goat anti-mouse IgG as the reagent control line (Fig. 1b). The latter (control line) was not included on the dipsticks used for the evaluation at the two international centers. The function of the control line is to ensure that the integrity of the conjugation of the anti-human IgG4 to the gold nanoparticles is intact upon storage. Since the rapid tests used in this study were from dipsticks and gold-conjugated antibody that were freshly prepared, the absence of the control line was not a problem. Furthermore, just before sending the dipsticks to the international centers, they were rigorously tested with a panel of positive and negative control sera to ensure that they worked well.

### Preparation of rAgB and lateral flow dipsticks

The rAgB was custom cloned (ProteoGenix, Schiltigheim, France) in pET32 expression vector (Novagen, Madison, WI, USA) and transformed into *Escherichia coli* strain C41 (DE3) (Lucigen, Middleton, WI, USA). The rAgB was expressed and subsequently purified according to a published method (24). The dipsticks and gold-conjugated IgG4 were prepared as previously described (25). Mouse anti-human IgG4 (Calbiochem, EMD Millipore, Billerica, MA, USA) was conjugated to colloidal gold particles (OD 8 at 530 nm) following a published method (26).

### Test procedure

**Fig. 2:**
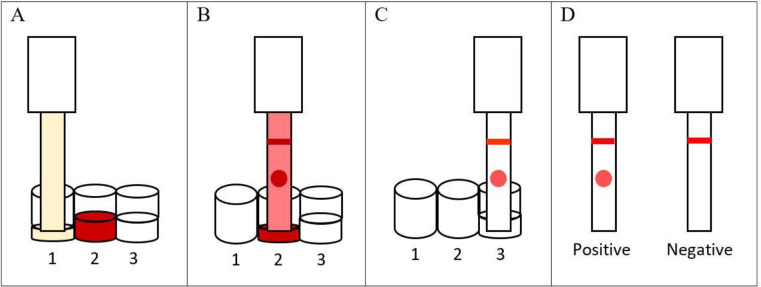
Hyd Rapid^TM^ test procedure. A: Seven μl of serum sample is diluted in 7 μl of Chase buffer (Reszon Diagnostics International, Selangor Darul Ehsan, Malaysia) in the first well; 20 μl of Chase buffer is added to a second well containing dried conjugate and is reconstituted after 30 seconds; 30 μl of wash buffer (PBS) is added to a third well. The dipstick is placed into the first well, and the diluted serum sample is allowed to flow up the dipstick until the well is dry. B: The dipstick is moved to the second well containing gold conjugate solution and allowed to flow up the dipstick until the well is dry. C: The dipstick is dipped into the third well and the wash buffer is allowed to flow up the dipstick until the background becomes clear. D: If a clear red dot and a red line are observed on the dipstick, the test is considered ‘positive’, and if a red dot does not appear while the red control line appears, the test is considered ‘negative’

The test procedure is briefly described in Fig. 2. The diagnostic performance of Hyd Rapid™ was determined using anonymized stored/banked serum samples from each centre. At KMU, the samples were all from Iranian patients.

Swiss TPH is a parasitology reference laboratory; thus, samples came from Swiss residents, migrants, and returning travellers from endemic countries.

The diagnostic sensitivity was assessed by testing sera from CE patients who had a clinical history and symptoms of the disease and were confirmed by one or both of surgery or imaging along with positive serology by Echinococcus ELISAs or EITB. The serum samples were collected before treatment or surgery. The diagnostic specificity was assessed by testing with serum samples from healthy individuals from non-echinococcosis endemic areas, healthy blood donors, or individuals with other infectious diseases.

## Results

The results of the evaluation at each diagnostic center are shown in Table 1. The in-house evaluation showed 95% diagnostic sensitivity and specificity. Cross-reaction were noted with 2 of 3 fascioliasis samples. The independent evaluation at KMU also showed 95% (18/19) sensitivity for CE detection. At Swiss TPH, the results showed a diagnostic sensitivity of 90% (9/10) for CE and 100% (n=10) for probable alveolar echinococcosis (AE); thus, the overall sensitivity for detection of echinococcosis (AE and CE) was 95% (19/20). However, Swiss TPH recorded 76% (22/29) diagnostic specificity, with cross-reactivity observed when tested using samples from patients with fascioliasis (4 of 11) and cysticercosis (3 of 9). If the results from fascioliasis and cysticercosis samples were excluded from USM and Swiss TPH, the diagnostic specificity at each centre was 100%. Among the sera from the 40 apparently healthy individuals from Kerman, 8 showed positive results with the Hyd Rapid™. Since Kerman is endemic for CE, they may have asymptomatic CE infections. Thus the results from the 40 serum samples from Kerman were not included in the diagnostic specificity determination.

**Table 1:** Summary of the evaluation results of the diagnostic sensitivity and specificity of Hyd Rapid™ test

***Institution***	***Reactivity of Hyd Rapid*™**				***Sensitivity***	Specificity^**^
	***CE***	***AE***	Other infections^*^	***Healthy***		
USM	20/21	-	0/28 (2/32)	0/11	95%	100% (95%)
Swiss TPH	9/10	10/10	0/4 (7/24)	0/5	95%	100% 76%
KMU	18/19	-	-	-^†^	95%	-
		Average	0/32 (9/56)	0/16	95%	100% (88%)

CE: Cystic Echinococcosis

AE: Alveolar Echinococcosis

USM: Universiti Sains Malaysia

Swiss TPH: Swiss Tropical and Public Health Institute, Switzerland

KMU: Kerman University of Medical Sciences, Kerman, Iran

* The reactivity with (and without) exclusion of cysticercosis and fascioliasis respectively

** The specificity with (and without) exclusion of cysticercosis and fascioliasis respectively

† Healthy individuals were from Kerman, a CE endemic area, thus were excluded since some of them may have asymptomatic CE

## Discussion

The 95% diagnostic sensitivity of the Hyd Rapid™ in detecting CE in this study was higher than in some other reports (12,27,28). In one report, 72% sensitivity in detecting CE was achieved using ELISA and HCF as antigen (29). An ELISA using recombinant AgB8/1was developed for CE detection, and reported the same sensitivity (72%) (28). IgG antibody detection assays have been developed for CE diagnosis based on the lateral flow format (15,28,30,31). They used native antigens B and 5, camel HCF and sheep HCF, respectively and all showed diagnostic sensitivity results of > 90%.

Evaluation of Hyd Rapid™ at the Swiss TPH clearly showed that the RDT did not differentiate CE from AE, with an overall diagnostic sensitivity of 95% for detecting both types of echinococcosis. The ability of Hyd Rapid™ to detect both CE and AE could make it useful as a screening test in areas where CE and AE are co-endemic, such as in parts of China (32). An immunochromatographic test based on HCF and rEm18 was evaluated to detect both CE and AE (33). The diagnostic sensitivity and specificity of the test was greater than 94% for both conditions.

At KMU, where the CE patients were confirmed as having active hydatid cyst lesions, Hyd Rapid^TM^ detected 100% of patients with liver CE and 89% of lung CE patients. Unfortunately, at USM and Swiss TPH, information on the cyst stage (active or inactive cyst) and cyst location was not available.

Currently, only a few RDTs for CE diagnosis are commercially available. A previous study compared three of the RDTs namely VIRapid® HYDATIDOSIS (based on purified Ag B and Ag 5; Vircell, Salamanca, Spain), Echinococcus Dot Immunogold Filtration Assay (DIGFA, based on purified cyst fluid, protoscolex antigen, antigen B and antigen Em2 of *E. multilocularis*; Unibiotest, Wuhan, China), and ADAMU-CE (based on recombinant Ag B1; ICST, Saitama, Japan) (34). The diagnostic sensitivities were reported to be 74%, 57%, and 72%, respectivity, while the specificities were 96%, 100%, and 72%, respectively. VIRapid® HYDATIDOSIS was deemed to show the best diagnostic performance. However, the diagnostic specificity testing in the study did not include sera from potentially cross-reactive diseases such as fascioliasis and cysticercosis.

Recently, Hyd Rapid™ was compared with VIRapid® HYDATIDOSIS (25). Hyd Rapid™ was positive in 79% of patients with active cysts (CE1, CE2, CE3a, CE3b) and 39% of patients with inactive cyst (CE4, CE5), and the overall diagnostic performance of Hyd Rapid™ was not statistically different (*P*=0.18) from that of VIRapid® HYDATIDOSIS (25). However, among CE1 patients, Hyd Rapid™ showed lower sensitivity (71.4%, n=7) compared to VIRapid® HYDATIDOSIS (100%). Both tests were 95% specific when tested with sera from individuals with no cysts (n=4) and patients with non-parasitic hepatic cysts (n=21) (25).

Since Kerman is a CE endemic area, asymptomatic CE infections were probably detected in the eight people with Hyd Rapid™ positive results among the 40 healthy individuals at KMU. Although animal fascioliasis has been reported in Kerman, there is no report of human infection (35,36). Thus the results from the eight individuals were unlikely due to co-infection with *Fasciola*. Since the samples were anonymised, they could not be contacted for further investigations.

In the present study, the overall diagnostic specificity of Hyd Rapid™ when tested with non-echinococcosis sera was 88%. The result was lower than the specificity of RDTs reported by Al-Sherbiny et al (15), Wang et al (30), and Gao et al (33); but higher than that reported by Sbihi et al (31). At Swiss TPH, Hyd Rapid™ cross-reacted with 36% (4/11) samples from fascioliasis patients and 33% (3/9) samples from cysticercosis patients. The finding is not surprising since cross-reactions with sera from both diseases have been reported in other studies on echinococcosis diagnostic assays (19,20,30,37–40). If these two types of samples were excluded from specificity calculation, 100% diagnostic specificity of Hyd Rapid™ was achieved. A comparison of diagnostic specificities among different assays is difficult unless the same panel of serum samples are used.

Another evaluation study on VIRapid® HYDATIDOSIS showed a specificity of 89%, including cross-reactivity with fascioliasis (4 of 8) and cysticercosis (2 of 3) (40). In yet another evaluation study of VIRapid® HYDATIDOSIS, a diagnostic specificity of 87.5% reported, and false positives reported with sera from 4 of 15 taeniasis patients (41). Thus, with regard to *Taenia* and *Fasciola* infections, VI-Rapid® HYDATIDOSIS showed the same specificity issue as Hyd Rapid™. Despite some cross-reaction with cysticercosis and fascioliasis, both VIRapid®HYDATIDOSIS and Hyd Rapid™ tests can still be considered useful as first-line diagnostic and screening tests for echinococcosis in endemic as well as non-endemic regions. In places where CE coexists with AE and/or fascioliasis and/or cysticercosis, a positive RDT result calls for further investigations. The final diagnosis can be made after a thorough evaluation of the patient’s imaging data, present illness and chief complaints, history (including travels), geographical origin, and other epidemiological information.

## Conclusion

Hyd Rapid™ has the potential for rapid diagnosis of echinococcosis, and it cannot differentiate CE from AE. Conversion of the RDT to a cassette format would make it physically more robust, especially for use in the field and remote areas. Further refinement of the RDT may increase its diagnostic sensitivity for CE1 patients and decrease the cross-reactivity with fascioliasis and cysticercosis sera. Regarding the latter, the addition of separate test dots/lines in the RDT that are specific for fascioliasis and cysticercosis may be useful in areas that are co-endemic for those diseases. Furthermore, a larger panel of serum samples from patients with different cyst stages and locations, and different countries would be necessary to confirm the diagnostic usefulness of Hyd Rapid™ for serodiagnosis of echinococcosis.

## Ethical considerations

Ethical issues (including plagiarism, informed consent, misconduct, data fabrication and/or falsification, double publication and/or submission, redundancy, etc.) have been completely observed by the authors.
